# Infective Endocarditis Risk After Invasive Dental Procedures

**DOI:** 10.1016/j.mayocpiqo.2025.100676

**Published:** 2025-11-11

**Authors:** Martin H. Thornhill, Peter B. Lockhart, Mark J. Dayer, Bernard D. Prendergast, Larry M. Baddour

**Affiliations:** a Unit of Oral & Maxillofacial Medicine, Surgery and Pathology, School of Clinical Dentistry, University of Sheffield, UK; b Department of Oral Medicine/Oral & Maxillofacial Surgery, Wake Forest University School of Medicine, Atrium Health - Carolinas Medical Center, Charlotte, NC; c Cardiovascular Research Institute, Mater Private Network, Dublin, Ireland, and Faculty of Health, University of Plymouth, UK; d Department of Cardiology, St Thomas’ Hospital and Cleveland Clinic, London, UK; e Division of Public Health, Infectious Diseases and Occupational Medicine, Departments of Medicine and Cardiovascular Medicine, Mayo Clinic College of Medicine and Science, Rochester, MN

## Abstract

**Objective:**

To quantify the risk of infective endocarditis (IE) following different invasive dental procedures in patients with cardiac risk factors that place them at low-risk, moderate-risk, or high-risk of developing IE.

**Patients and Methods:**

The linked IBM MarketScan administrative databases were used to integrate deidentified patient-level health data for all enrollees over 18 years of age with employer-provided commercial/Medicare-supplemental medical and dental coverage, or Medicaid benefits, with more than 16 months of data from May 1, 2007, to August 31, 2015.

**Results:**

In the resulting 9.6 million patient cohort, IE incidence in the 4-months following 53.6 million invasive dental procedures was quantified. In high-risk individuals (e.g. previous IE, prosthetic/repaired heart valves, or cyanotic congenital heart disease), IE incidence in the 4 months following an IDP was 2195 IE cases/million procedures - ∼125 times higher than in low-risk (OR 126.3; 95% CI, 113.5-140.6; *P*<.001). The IE-risk was even greater following extractions (incidence 8680 IE cases/million extractions, OR 171.4; 95% CI, 136.7-214.8; *P*<.001) or other oral surgical procedures (incidence 13,458 IE cases/million procedures; OR 245.5; 95% CI, 165.1-365.1; *P*<.001). Moderate-risk individuals were at significantly lower IE-risk, and low-risk individuals were at negligible risk.

**Conclusion:**

The risk of IE was high in high-risk individuals following all types of IDP (particularly following extractions and other oral surgical procedures) and vastly exceeded the risk of adverse drug reactions following antibiotic prophylaxis. Our data therefore support guidance recommending high-risk individuals receive antibiotic prophylaxis and provide quantitative information concerning the IE-risk that can be used to educate and obtain informed consent from patients.

International guidelines, including the American Heart Association (AHA) and European Society for Cardiology guidelines, recommend antibiotic prophylaxis (AP) before invasive dental procedures (IDPs) to reduce the likelihood of infective endocarditis (IE) in those at high IE-risk.^1^^,^^2^ To date, the risk posed by different IDPs has been derived from studies that investigated the frequency with which bacteremia occurred following different IDPs. These relied on the hypothesis that a bacteremia is necessary for IE to develop, and any procedure associated with a considerable likelihood of bacteremia could, therefore, pose a threat of IE. Although many bacteremia studies have been performed, permitting the identification of those IDPs most likely to pose a threat,^3^ bacteremia is only a surrogate measure for the risk of developing IE and tells us nothing about how this risk varies in different patient cohorts.

A relevant bacteremia results from IDPs in most individuals. However, the IE-risk posed by this bacteremia is extremely small in someone with no cardiac risk factors for IE (i.e. someone at low IE-risk). It may be much greater in someone at high-risk for IE. Quantification of the real risk posed by IDPs to individuals at different levels of IE-risk is therefore important. It is particularly important for patients at increased IE-risk, who need to understand the risks involved when providing informed consent for any proposed IDPs and if they wish to have AP to reduce the risk.

Although bacteremia studies can be performed with small numbers of individuals, much larger studies are needed to quantify the incidence of IE following IDPs since IE incidence is low. We therefore quantified the incidence of IE occurring soon after IDPs in a large US patient cohort comprising patients with different levels of risk for developing IE.

## Patients and Methods

### Study Design

The study was a retrospective cohort study of patients with employer-provided commercial or Medicare-supplemental medical, dental, and prescription benefits insurance cover and Medicaid patients from states where adults receive basic medical, dental, and prescription benefits cover. Data were obtained from the linked IBM MarketScan administrative databases, which were used to integrate deidentified patient-level health data for all enrollees (see Supplemental Appendix, available online at http://www.mcpiqojournal.org).^4^, ^5^, ^6^ The study quantified the incidence of IE occurring within 4 months of an IDP, in patients at high-risk, moderate-risk, and low-risk of developing IE. The study adhered to Strengthening the Reporting of Observational studies in Epidemiology guidelines for cohort studies.^7^

### Inclusion/exclusion Criteria

All enrollees with employer-provided commercial, Medicare-supplemental, or Medicaid, medical, dental, and prescription benefits cover aged ≥18 years and with >16 months of linked data for the period from May 1, 2007, to August 31, 2015 were included in the study.

### Ethics

The MarketScan databases were not subject to United States institutional review board review since they are statistically deidentified (in compliance with the US 1996 Health Insurance Portability and Accountability Act) and meet Health Insurance Portability and Accountability Act limited-use criteria.^8^

### IE Admissions and Risk Stratification

Databases were interrogated using International Classification of Disease (ICD)-9 primary or secondary discharge diagnostic codes 421.0, 421.1 or 421.9 to identify all IE-related hospital admissions. Previously described methods were used to ensure single counting of continuous IE episodes.^9^ New episodes were distinguished from readmissions by excluding IE admissions <6 months apart.^4^^,^^10^ Although misclassification of cases may occur in administrative databases, a recent study using ICD-10 codes (equivalent to the ICD-9 codes used in this study) reported high sensitivity and specificity for the accurate identification of modified Duke criteria-positive IE cases.^11^

Using available medical records data back to January 2000, ICD-9 or current procedural terminology diagnostic/procedural codes were used to identify individuals at high or moderate IE-risk based on AHA definitions (Table 1, Supplemental Tables 1 and 2, available online at http://www.mcpiqojournal.org).^2^^,^^12^^,^^13^ All remaining individuals were considered at low IE-risk.

**Table 1:** Cardiac Conditions Used to Classify Individuals as High or Moderate IE-risk^a^^,^^b^

**High IE-Risk**
Previous history of infective endocarditis
Prosthetic heart valve (including transcatheter devices)
Prosthetic material used for valve repair (including annuloplasty and transcatheter valve procedures using prosthetic material)
Unrepaired cyanotic congenital heart disease
Congenital heart disease treated with palliative shunts or conduits
Congenital heart defect completely repaired using surgical or transcatheter prosthetic material or device (for 6 months following the procedure)
**Moderate IE-Risk**
Rheumatic heart disease
Nonrheumatic valve disease (including mitral valve prolapse)
Congenital valve anomalies (including aortic stenosis)
Hypertrophic cardiomyopathy

a IE, infective endocarditis.

b Adapted from the American Heart Association guidelines.^2^^,^^12^^,^^13^ More extensive details concerning diagnoses and procedures (including the relevant ICD-9-CM diagnosis and procedure codes and current procedural terminology procedure codes) are provided in Supplemental Tables 1 & 2.

### Invasive Dental Procedures

The American Dental Association Common Dental Terminology codes^14^ and ICD-9 procedural codes^15^ were used to identify IDPs as defined by the AHA guidelines, i.e., All dental procedures that involve manipulation of gingival tissues or the periapical region of teeth or perforation of the oral mucosa (excepting anesthetic injections through noninfected material).^2^^,^^13^ When a treatment involved multiple visits, each visit was evaluated separately for use of AP and procedures performed. IDPs were also sub-analyzed using codes specific for extractions (surgical and nonsurgical), endodontic procedures, oral surgical procedures (including biopsies, implant, and periodontal surgery), restorative procedures likely to have involved gingival manipulation, and different types of professional dental cleaning, scaling, and periodontal examination procedures (Supplemental Tables 3-5, available online at http://www.mcpiqojournal.org). The professional dental cleaning, scaling and periodontal examination procedures analyzed separately included basic periodontal screening exam (BPE), professional dental cleaning (supragingival scale and polish), and subgingival scaling or root planing.

### Incidence of IE following IDPs

Subjects were stratified according to IE-risk (high, moderate, or low/unknown) and followed until study completion, expiry of linked data, or death. The incidence of IE in the 4 months following IDPs was quantified. IE incidence was compared between different IE-risk-groups and different types of dental procedure. Crude incidence was adjusted for differences in age, sex, and Charlson Comorbidity Index among groups.^16^ Because AP cover was used in this population, we quantified AP cover of IDP in high-risk, moderate-risk, and low-risk patient groups. Data on IE incidence following IDP were corrected to account for AP use, since previous studies have shown high AP efficacy in preventing IE following IDPs in these populations.^5^^,^^6^

Many studies have used a 3 month to 4 month window to investigate the association between potential causes of IE and IE development based on incubation time and time between first symptom development and eventual IE diagnosis.^17^ Several studies have also shown that disease onset and diagnosis are more likely to be delayed in IE cases caused by oral viridans group streptococci compared to cases caused by staphylococci or other more aggressive organisms.^17^^,^^18^ Hence, we chose a 4 month window between the IDP and IE diagnoses to identify IE cases most likely to be associated with IDP.

### Statistics

To address the rare outcome of interest (2,634 IE cases within 4 months of a dental procedure in a population of 9,630,162), Firth logistic regression, a penalized-likelihood statistical method, was applied. This method was introduced to address the possibility of rare outcomes causing small sample size bias (particularly in some sub-analyses) when using traditional maximum likelihood logistic regression, which can lead to the non-convergence of regression estimates.^19^^,^^20^ We set a *P*<.05 criterion for determining significance, but we first applied a Bonferroni correction to the *P* values to account for situations where multiple comparisons were performed.

## Results

The study cohort consisted of 9.63 million individuals, of whom, 7.95 million had employer-provided Commercial/Medicare-Supplemental health cover, and 1.68 million were Medicaid patients.

The incidence of IE, stratified by IE-risk, within 4 months of the 53,692,217 different IDPs performed on this cohort of patients, was quantified. Demographic details and the number of different IDPs in those with employer-provided commercial/Medicare coverage or Medicaid cover are shown in Table 2. Among the entire population, 44,983 (0.05%) were at high-risk of IE, 650,428 (6.8%) were at moderate-risk, and 8,934,751 (92.8%) were at low/unknown risk. Prescribing data for these populations demonstrated that AP was used before IDP in 32.1% of high-risk individuals, 9.6% of moderate-risk individuals received AP, and 3% of low-risk individuals. Differences in AP prescribing rates between patients with Medicaid and commercial/Medicare-supplemental cover are shown in Table 2.

**Table 2:** Demographic and Descriptive Data for the Medicaid and Commercial/Medicare-Supplemental Populations

Population data	Medicaid	Commercial/Medicare-supplemental	All
Population (Total)	1,678,190 (100%)	7,951,972 (100%)	9,630,162 (100%)
Age 18-34	1,045,184 (62.3%)	2,435,930 (30.6%)	3,481,114 (36.2%)
Age 35-44	264,375 (15.8%)	1,573,862 (19.8%)	1,838,237 (19.1%)
Age 45-54	190,581 (11.4%)	1,794,556 (22.6%)	1,985,137 (20.6%)
Age 55-64	117,443 (7.0%)	1,473,689 (18.5%)	1,591,132 (16.5%)
Age 65+	60,607 (3.6%)	673,935 (8.5%)	734,542 (7.6%)
Male	501,408 (29.9%)	3,691,739 (46.4%)	4,193,147 (43.5%)
Female	1,176,782 (70.1%)	4,260,233 (53.6%)	5,437,015 (56.5%)
CCI (previous 12 m)			
0	1,396,086 (83.2%)	6,592,951 (82.9%)	7,989,037 (83.0%)
1	172,541 (10.3%)	851,964 (10.7%)	1,024,505 (10.6%)
2	47,651 (2.8%)	287,476 (3.6%)	335,127 (3,5%)
3+	61,912 (3.7%)	219,851 (2.8%)	281,763 (2.9%)
Cardiac risk			
High risk	8210 (0.5%)	36,773 (0.5%)	44,983 (0.05%)
Moderate risk	86,739 (5.2%)	563,689 (7.1%)	650,428 (6.8%)
Low/unknown risk	1,583,241 (94.3%)	7,351,510 (92.5%)	8,934,751 (92.8%)
Invasive dental procedure (IDP) Data			
All IDPs	3,833,074 (100%)	49,859,143 (100%)	53,692,217 (100%)
Basic periodontal screening exam (BPE)	2045 (0.05%)	373,404 (0.8%)	375,449 (0.7%)
All dental prophylaxis and scaling procedures	1,561,767 (40.8%)	35,629,327 (71.5%)	37,191,094 (69.3%)
Dental prophylaxis	1,379,161 (36.0%)	30,498,537 (61.2%)	31,877,698 (59.4%)
Subgingival scaling and/or root planing	182,606 (4.8%)	5,130,790 (10.3%)	5,313,396 (9.9%)
Extractions	1,091,431 (28.5%)	2,122,760 (4.3%)	3,214,191 (6.0%)
Endodontic procedures	114,867 (3.0%)	1,465,025 (2.9%)	1,579,892 (2.9%)
Oral surgery procedures	99,946 (2.6%)	731,689 (1.5%)	831,635 (1.6%)
Restorative procedures	963,018 (25.1%)	9,536,938 (19.1%)	10,499,956 (19.6%)
Antibiotic prophylaxis use			
AP cov. IDPs - HR	3470 (25.9%)	59,045 (32.6%)	62,515 (32.1%)
AP cov. IDPs - MR	14,222 (10.5%)	272,133 (9.5%)	286,355 (9.6%)
AP cov. IDPs - LR	97,673 (3.8%)	1,047,154 (2.9%)	1,143,827 (3.0%)
Infective endocarditis (IE)			
IE within 4 mo of procedure	577 (0.015%)	2057 (0.004%)	2634 (0.005%)

Abbreviations: AP, antibiotic prophylaxis; CCI, Charlson comorbidity index score; cov, cover; HR, high-risk; IDPs, invasive dental procedures; IE, infective endocarditis; LR, low-risk; m, months; MR, moderate-risk; proc, procedures; suppl, supplemental

### Incidence of IE Following IDP Adjusted for AP use

**Figure 1:**
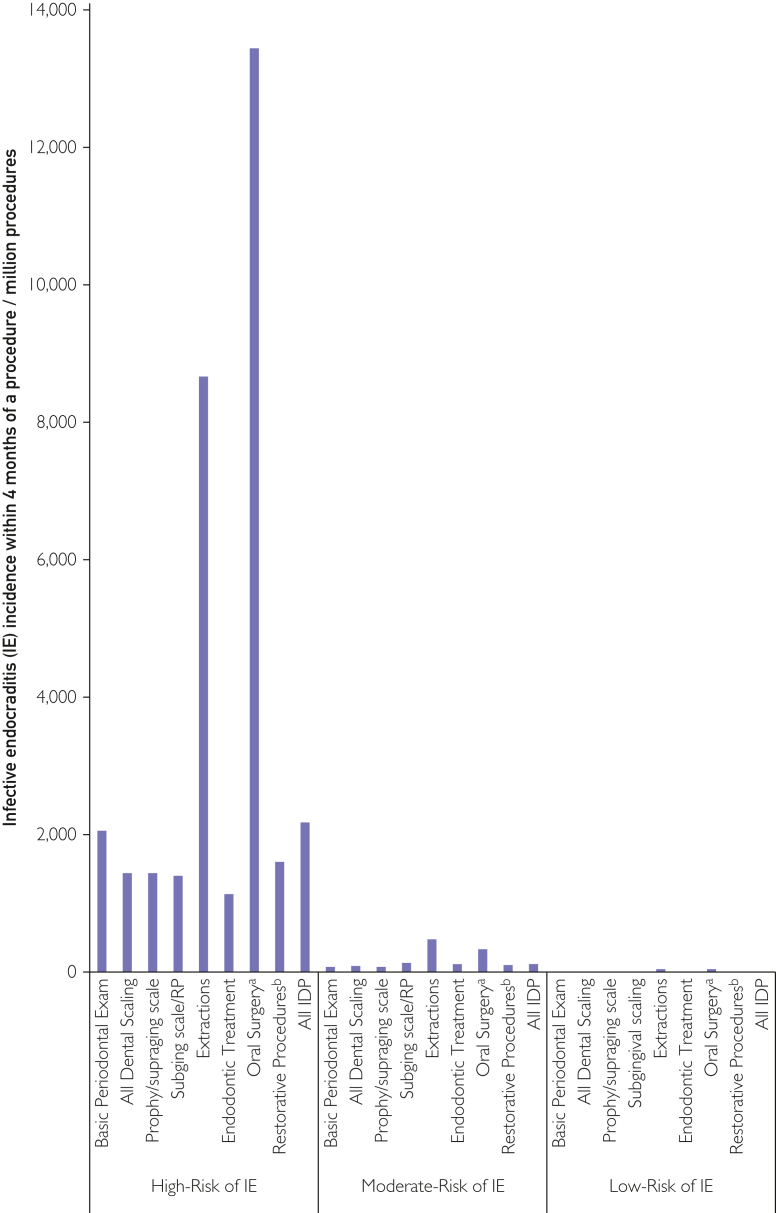
Infective endocarditis (IE) incidence within 4-months of invasive dental procedures (IDPs) in patients at high-, moderate- or low-risk of IE. IDP, invasive dental procedures; IE, infective endocarditis; Prophy, dental prophylaxis; RP, root planning; Scale, scaling; Subging, subgingival; Supraging, supragingival. ^a^Includes biopsies, implant and periodontal surgery. ^b^Restorative procedures likely to involve gingival manipulation.

**Figure 2:**
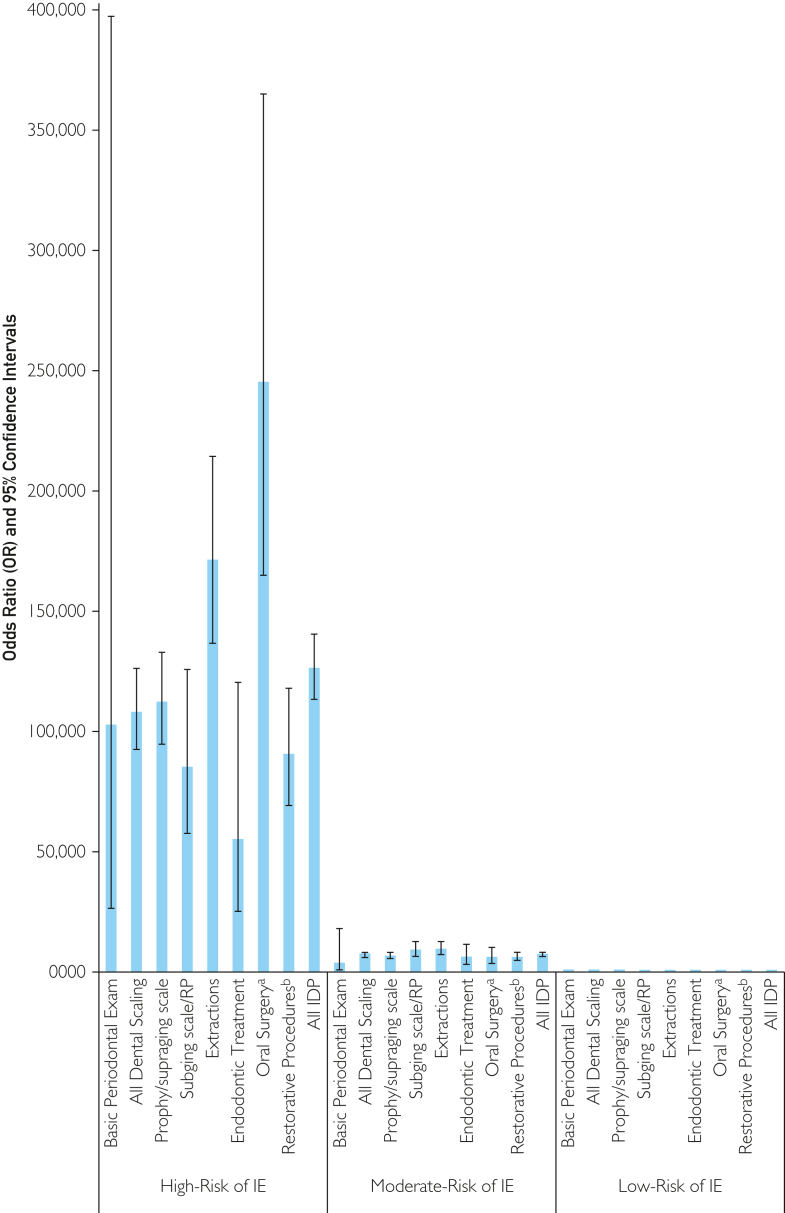
Odds of infective endocarditis (IE) following invasive dental procedures (IDPs) in individuals at high- or moderate-risk of IE compared to those at low-risk. IDP, invasive dental procedures; IE, infective endocarditis; Prophy, dental prophylaxis; RP, root planning; Scale, scaling; Subging, subgingival; Supraging, supragingival. ^a^Includes biopsies, implant and periodontal surgery. ^b^Restorative procedures likely to involve gingival manipulation.

The purpose of AP is to reduce the risk of developing IE following IDP and recent studies have shown the efficacy of this approach, particularly in high-risk individuals.^5^^,^^6^^,^^21^ Because a relevant proportion of these individuals received AP cover for IDP, it is likely that the raw data considerably underestimated the incidence of IE following IDP. We therefore adjusted the raw incidence data (Supplemental Table 6, available online at http://www.mcpiqojournal.org) to address this issue, using the rates of AP prescribing for each patient cohort and risk category (Figures 1 and 2; Tables 2 and 3,).

**Table 3:** IE Incidence Within 4 months of Different Invasive Dental Procedures (IDPs) Corrected for Antibiotic Prophylaxis Use

Type of procedure	Patients with commercial/medicare-supplemental cover										
	High-Risk of IE				Moderate-Risk of IE				Low-Risk of IE		
	Proc (n)	IE (n)	IE/million proc.	High vs low OR, 95% CI, *P*	Proc. (n)	IE (n)	IE/million proc.	Mod vs Low OR, 95% CI, *P*	Proc. (n)	IE (n)	IE/million proc
Basic periodontal screening exam (BPE)	1432	3	2095	103.764 (26.806-401.666), *P*<.001	25,981	2	77	3.805 (0.790-18.318), *P*=.047	345,991	7	20
All dental prophylaxis/scaling	161,839	226	1396	105.520 (89.853-123.918), *P*<.001	2,567,587	244	95	7.172 (6.131-8.388), *P*<.001	32,899,901	436	13
Dental prophylaxis	136,574	194	1420	110.786 (93.049-131.904), *P*<.001	2,168,556	186	86	6.752 (5.662-8.053), *P*<.001	28,193,407	362	13
Subgingival scaling/root planning	25,265	31	1227	78.133 (51.363-118.856), *P*<.001	399,031	58	145	9.246 (6.556-13.038), *P*<.001	4,706,494	74	16
Extractions	11,483	83	7228	214.332 (155.052-296.277), *P*<.001	168,278	58	345	10.150 (7.133-14.444), *P*<.001	1,942,999	66	34
Endodontic treatment	6621	7	1057	49.071 (21.489-112.056), *P*<.001	113,780	13	114	5.298 (2.754-10.192), *P*<0.0001	1,344,624	29	22
Oral surgery^a^	3628	38	10,474	369.570 (212.866-641.635), *P*<.001	64,663	14	217	7.561 (3.791-15.080), *P*<.001	663,398	19	29
Restorative procedure^b^	46,143	69	1495	91.879 (68.926-122.475), *P*<.001	717,454	72	100	6.158 (4.639-8.174), *P*<.001	8,773,341	143	16
Total	231,146	426	1843	121.254 (107.488-136.783), *P*<.001	3,657,743	403	110	7.236 (6.402-8.180), *P*<.001	45,970,254	700	15

Type of Procedure	Patients with Medicaid Cover										
	High-Risk of IE				Moderate-Risk of IE				Low-Risk of IE		
	Proc. (n)	IE (n)	IE/million proc.	High vs Low OR, 95% CI, *P*	Proc.(n)	IE (n)	IE/million proc.	Mod vs Low OR, 95% CI, *P*	Proc. (n)	IE (n)	IE/million proc.
Basic periodontal screening exam (BPE)	22	0	0	nc	89	0	0	nc	1934	0	0
All dental prophylaxis/scaling	7091	19	2679	154.478 (85.459-279.236), *P*<.001	59,698	14	235	13.487 (7.043-25.830), *P*<.001	1,494,978	26	17
Dental prophylaxis	6192	13	2099	146.361 (72.253-296.481), *P*<.001	51,186	10	195	13.594 (6.321-29.235), *P*<.001	1,321,783	19	14
Subgingival scaling/root planning	899	6	6674	166.234 (55.756-495.615), *P*<.001	8512	4	470	11.632 (3.404-39.742), *P*<.001	173,195	7	40
Extractions	6029	69	11,445	137.899 (100.274-189.641), *P*<.001	72,861	60	823	9.817 (7.054-13.663), *P*<.001	1,012,541	85	84
Endodontic treatment	403	1	2481	275.908 (17.228-4,418.716), *P*<.001	3548	2	564	62.558 (5.671-690.061), *P*<.001	110,916	1	9
Oral surgery^a^	756	21	27,778	115.034 (63.385-208.768), *P*<.001	7015	11	1568	6.323 (3.081-12.977), *P*<.001	92,625	23	248
Restorative procedure^b^	3714	11	2962	94.651 (47.248-189.613), *P*<.001	35,248	12	340	10.851 (5.537-21.266), *P*<.001	924,056	29	31
Total	18,015	121	6717	149.956 (118.510-189.746), *P*<.001	178,459	99	555	12.309 (9.591-15.797), *P*<.001	3,637,050	164	45

Type of Procedure	All Patients										
	High-Risk of IE				Moderate-Risk of IE				Low-Risk of IE		
	Proc. (n)	IE (n)	IE/million proc.	High vs Low OR, 95% CI, *P*	Proc. (n)	IE (n)	IE/million proc.	Mod vs Low OR, 95% CI, *P*	Proc. (n)	IE (n)	IE/million proc.
Basic periodontal screening exam (BPE)	1454	3	2063	102.762 (26.547-397.781), *P*<.001	26,070	2	77	3.813 (0.792-18.357), *P*<.048	347,925	7	20
All dental prophylaxis/scaling	168,930	245	1450	108.127 (92.604-126.253), *P*<.001	2,627,285	258	98	7.311 (6.278-8.515), *P*<.001	34,394,879	462	13
Dental prophylaxis	142,766	207	1450	112.484 (94.964-133.236), *P*<.001	2,219,742	196	88	6.841 (5.758-8.127), *P*<.001	29,515,190	381	13
Subgingival scaling/root planning	26,164	37	1414	85.312 (57.814-125.891), *P*<.001	407,543	62	152	9.166 (6.585-12.759), *P*<.001	4,879,689	81	17
Extractions	17,512	152	8680	171.369 (136.746-214.757), *P*<.001	241,139	118	489	9.582 (7.531-12.192), *P*<.001	2,955,540	151	51
Endodontic treatment	7024	8	1139	55.322 (25.354-120.712), *P*<.001	117,328	15	128	6.204 (3.338-11.530), *P*<.001	1,455,540	30	21
Oral surgery^a^	4384	59	13,458	245.543 (165.117-365.144), *P*<.001	71,678	25	349	6.280 (3.828-10.304), *P*<.001	756,023	42	56
Restorative procedure^b^	49,857	80	1605	90.611 (69.590-118.151), p<.001	752,702	84	112	6.293 (4.847-8.168), *P*<.001	9,697,397	172	18
Total	249,161	547	2195	126.324 (113.487-140.614), *P*<.001	3,836,202	502	131	7.514 (6.732-8.388), *P*<.001	49,607,304	864	17

Abbreviations: Cl, confidence limits; IE, infective endocarditis; high vs low, odds (with 95% Cl) of developing IE comparing high-risk with low-risk individuals; mod vs low, odds (with 95% Cl) of developing IE comparing moderate-risk with low-risk individuals; OR, odds ratio; *P*, p value; proc, procedures.

a Includes biopsies, implant and periodontal surgery.

b Restorative procedures likely to involve gingival manipulation.

The corrected overall incidence of IE in the 4 months following an IDP was 2195 per million procedures in high-risk individuals. This was significantly higher than in moderate-risk (131 per million procedures; OR 16.8, 95% CI 14.9-19.0, *P*<.001) or low-risk (17 per million procedures; OR 126.3, 95% CI 113.5-140.6, *P*<.001) individuals. Furthermore, the corrected incidence of IE was highest in those at high IE-risk for all types of IDP investigated. But it was particularly high following dental extractions (8680 IE cases per million procedures in those at high-risk vs 51 IE cases per million procedures in those at low-risk; OR 171.4, 95% CI 136.7-214.8, *P*<.001), and oral surgery procedures (13,458 IE cases per million procedures vs 56 per million; OR, 245.5; 95% CI, 165.1-365.1; *P*<.001)—including biopsies, implant surgery, and periodontal surgery.

For dental prophylaxis/supragingival scaling and subgingival scaling/root planing procedures in high-risk individuals, the incidence of IE was high (1450 IE cases per million procedures and 1414 IE cases per million procedures, respectively), with no significant difference between these procedures.

## Discussion

Bacteremia—the entry of bacteria into the circulation—is a prerequisite for the development of IE. Multiple studies dating back to the early 1950s have shown that IDPs can result in bacteremia with oral species (particularly oral viridans group streptococci and *Haemophilus*, *Aggregatibacter*, *Cardiobacterium*, *Eikenella*, and *Kingella* species) that can potentially cause IE.^3^ However, the likelihood of developing IE following an IDP also depends on the susceptibility of the individual. In low-risk patients with no predisposing cardiac risk factors, the endothelial lining of the heart is resistant to infection arising from the frequent bacteremia caused by daily activities, such as chewing or tooth brushing, as well as the larger bacteremia that may result from IDPs. In contrast, chronic or recurrent endothelial damage may result in high-risk patients with congenital or acquired heart conditions, previous IE or the presence of intracardiac prosthetic material (Table 1),^22^^,^^23^ resulting in the release of inflammatory cytokines and tissue factors, exposure of subendothelial fibronectin, and formation of platelet-fibrin thrombus that facilitates bacterial adherence and colonization.^22^^,^^24^

Bacteremia studies can identify dental procedures that may cause a bacteremia but tell us little about the risk of developing IE following an IDP. Large population studies are required for this purpose and enable us to determine the incidence of IE immediately following IDPs in patients at high-risk, moderate-risk, and low-risk of IE. Our findings are important for patients and clinicians in obtaining informed consent to perform IDP and in evaluating the risks and benefits of AP.

This study was performed in the United States using data from patients with employer-provided medical, dental, and prescription benefits insurance cover, employer-provided Medicare-supplemental cover (for older patients); or Medicaid cover, where available. Thus, it provided important representative data from a broad spectrum of different health care systems and patient types (Table 3). Because the data were from the United States, many patients (particularly those at high-risk) would have received AP before IDPs as recommended by AHA guidelines.^2^ Of note, AP has been shown to be highly effective in reducing the incidence of IE following IDP in patients at high-risk of IE^5^^,^^6^ and the raw data are therefore likely to have significantly underestimated the incidence of IE (Supplemental Table 6). The availability of the percentage of patients in each risk group who received AP before IDP enabled us to correct for this.

The corrected data (Figures 1 and 2; Table 3,) showed that, while not zero, the incidence of IE in the 4 months following IDPs was low (17/million procedures) in individuals at low IE-risk. Given that the incidence of adverse drug reactions (ADR) rate is only 22.6/million prescriptions (none fatal) for amoxicillin AP and 162/million prescriptions (including 13 fatal) for clindamycin AP,^25^ risk posed by AP is likely to outweigh any IE prevention benefit in those at low IE-risk. The incidence of IE in the 4 months following an IDP was around 7.5 times higher (131/million procedures) in those at moderate IE-risk and any benefit from AP would be more marginal compared to the risk of ADR and may not be cost-effective,^26^ – particularly when incorporating the possibility of promoting antibiotic resistance.^27^ However, it is possible that the benefits of AP could outweigh the risks in particular moderate-risk patients such as those with multiple cardiac risk conditions, immunodeficiency/immunosuppression, poor oral hygiene, or need for particularly invasive dental procedures, eg, difficult or multiple extractions or more invasive oral surgical procedures.

In contrast, the incidence of IE in the 4 months following IDP in those at high IE-risk was 2195 IE cases/million procedures (126 times higher than that at low-risk). In this scenario, the risk posed by IE (which still has a ∼30% first-year mortality) is likely to significantly outweigh the risk posed by AP and remain cost-effective.^26^

The incidence of IE in high-risk individuals was highest following oral surgical procedures (including biopsy, implant, and periodontal surgery) at 13,458 IE cases/million procedures (almost 250 times higher than the incidence of IE following these procedures in those at low IE-risk). This incidence was significantly higher than following extractions (8680 IE cases/million procedures, *P*=.002; 171 times higher than the risk in those at low IE-risk).

The risk of IE following all other IDPs in those at high IE-risk was significantly lower than following oral surgical procedures (*P*<.0007) or extractions (*P*<.0067). Even though there were slight differences in IE incidence following these procedures (basic periodontal screening examination, supragingival scale and polish, subgingival, scaling/root planing, and endodontic or restorative dental procedures), none of these differences were statistically significant. Nonetheless, the odds of developing IE following any of these procedures were 55-112 times higher than when they were performed in low-risk individuals (Table 3). Although current ESC and AHA guidelines recommend that high-risk individuals receive AP before all IDPs investigated in this study,^1^^,^^2^ some commentators have questioned whether a BPE, or professional dental cleaning (supragingival scale and polish) are as invasive as subgingival scaling and root planing, and have advised against AP cover of these procedures.^28^ The present data reported that the risk of developing IE following a BPE or a professional dental cleaning is as high as that from subgingival scaling and root planning and other IDPs for which AP is recommended.

Although the distribution of IE-risk was similar in both study cohorts, the risk of developing IE following IDPs was higher in the Medicaid population. Although differences in general and dental health, access to care,^29^^,^^30^ and less frequent use of AP may partly explain this nearly 4-fold difference (6717 vs 1843 cases/million procedures, respectively), the number and type of IDPs performed in these 2 populations may also be responsible. Extractions accounted for 28.5% of all IDPs performed in Medicaid patients, but only 4.3% of those in the Commercial/Medicare cohort. Conversely, dental prophylaxis and scaling procedures accounted for 71.5% of IDPs in Commercial/Medicare patients but only 40.8% in the Medicaid cohort. These findings suggest reduced emphasis on preventive dental care and oral hygiene in Medicaid patients and a higher use of extractions. This difference in the risk of developing IE in distinct health care populations should sound an alarm to health care providers and public health authorities in the United States.

The quantitative data from this study provides important new information that a broad array of health care workers, including cardiologists, primary care clinicians, infectious disease specialists, cardiovascular surgeons, and dentists can use to advise patients concerning the risk of IE posed by different IDPs, direct decisions concerning the use of AP, and contribute to discussions at the time of obtaining informed consent.

### Study Limitations

Although we corrected for age, sex, and Charlson Comorbidity Index differences when comparing IE incidence in the cohort study, uncorrected differences or unmeasured confounders may have influenced the outcome.

We have previously validated the methodology that was used to determine if dental procedures were performed under AP cover or not and reported high sensitivity and specificity for the correct identification of whether antibiotics were used for IE prophylaxis or the treatment of dentoalveolar infections.^31^ However, we may have underestimated the use of AP (and therefore the incidence of IE following IDPs) because sensitivity was less than specificity. In other words, the true incidence of IE in high-risk individuals is likely to be higher than our corrected estimates.

## Conclusion

We have shown that the risk of developing IE in the 4 months following IDPs is around 125 times greater in those at high IE-risk, as compared with that in individuals at low IE-risk, and that risk is even higher in those undergoing dental extractions (nearly 175 times higher) or other oral surgical procedures (nearly 250 times higher). IE is fatal within a year of diagnosis in ∼30% of patients.^22^ and the risk of developing or dying from IE following an IDP is greater than the risk of developing an ADR when taking AP for IE prevention. Data from the current investigation strongly support the European Society for Cardiology and AHA recommendation that high-risk individuals should receive AP cover before IDPs, quantify the risk posed by IDPs in those at high IE-risk, and can be used to guide discussions with patients when obtaining informed consent.

## Potential Competing Interests

Drs Thornhill and Lockhart received support from the Delta Dental Research and Data Institute for the submitted work. None of the other authors reports a financial relationship in the previous three years with companies that might have an interest in the submitted work. Dr Dayer reports payments from Medscape for article writing and support from Abbott for attending meetings, both of which were unconnected to the submitted work. Dr Prendergast is a Board member and partner in CERC (a clinical research organization), and reports lecture fees from Edwards Lifesciences and Polares medical plus consultancy fees while serving on Research Advisory Boards for Valvosoft and Medtronic (unconnected to the submitted work). Dr Baddour has received royalty payments (authorship duties) from UpToDate, Inc.

Dr Prendergast acted as review coordinator for the 2023 ESC guidelines on the management of infective endocarditis while Drs Baddour and Thornhill were reviewers for these guidelines. Drs Baddour and Lockhart were members of the American Heart Association Committee on Rheumatic Fever, Endocarditis and Kawasaki Disease and were involved in drafting the 2021 American Heart Association guidelines on the prevention of infective endocarditis. Dr Dayer was a consultant to the review committee that produced the 2015 update to NICE clinical guideline 64 on prophylaxis against infective endocarditis.

## Ethics Statement

The Ethics statement was listed as the third category in the Patients and Methods section.
