# Supplementary material for: Infective Endocarditis Risk After Invasive Dental Procedures
Source: Mayo Clin Proc Innov Qual Outcomes. 2025 Nov 11;9(6):100676. doi: 10.1016/j.mayocpiqo.2025.100676 (PMC12650783; doi:10.1016/j.mayocpiqo.2025.100676)
Supplement: Supplementary Material [file mmc1.docx]

Supplementary Appendix:

Infective endocarditis risk following invasive dental procedures

Martin H. Thornhill, MBBS, BDS, PhDa, Peter B. Lockhart, DDSb, Mark J. Dayer, MBBS, PhDc, Bernard D. Prendergast, BM, BS, DMd, Larry M. Baddour, MDe.

aUnit of Oral & Maxillofacial Medicine, Surgery and Pathology, School of Clinical Dentistry, University of Sheffield, Sheffield, UK; bDepartment Oral Medicine/Oral & Maxillofacial Surgery, Atrium Health - Carolinas Medical Center, Charlotte, NC; cCardiovascular Research Institute, Mater Private Network, Dublin, Ireland, and Faculty of Health, University of Plymouth, Plymouth, UK; dDepartment of Cardiology, St Thomas’ Hospital and Cleveland Clinic, London, UK; eDivision of Infectious Diseases, Departments of Medicine and Cardiovascular Medicine, Mayo Clinic College of Medicine, Rochester, MN.

Index:

Page

[Abbreviations 3](#_TOC_250001)

[Supplementary-Methods 4](#_TOC_250000)

Supplementary Appendix Tables: 6

Table 1. ICD-9-CM Diagnosis and Procedure Codes, and CPT Outpatient Procedure 6

codes used to identify ‘high-risk’ cardiac conditions.

Table 2. ICD-9-CM Diagnosis and Procedure Codes, and CPT Outpatient Procedure codes 10

used to identify ‘moderate-risk’ cardiac conditions.

Table 3. Examples of invasive-dental procedures (IDPs) 12

Table 4. CDT and ICD-9 invasive dental procedure (IDP) codes. 13

Table 5. Definitions for ADA Procedure Codes used in this study 14

Table 6. Unadjusted (raw) IE incidence within 4 months of different invasive dental

procedures (IDP) 22

Supplementary References: 25

# Abbreviations:

ADA = American Dental Association AHA = American Heart Association AP = Antibiotic prophylaxis

CCI = Charlson comorbidity index

CPT = Current procedural terminology CDT = Common dental terminology ESC = European Society of Cardiology

HIPAA = Health Insurance Portability and Accountability Act ICD = International Classification of Disease

IDP = Invasive dental procedures IE = Infective endocarditis

IRB = Institutional review board

STROBE = Strengthening the Reporting of Observational Studies in Epidemiology reporting guidelines for cohort studies

US = United States of America

# Supplementary-Methods:

*Data Source:*

The IBM® MarketScan® databases integrate de-identified patient-level health-data across a series of healthcare related databases. These databases are now owned and managed by Merative®. We studied the Multi-State Medicaid (basic health cover provided in some states for those without medical insurance) database and, for comparison, linked data from the employer-funded Commercial (private health insurance cover provided mainly by employers as a benefit for their employees), Medicare-Supplemental (top up health insurance cover provided by employers for their retirees to improve the basic cover provided by Medicare), prescription benefits and Dental (insurance cover for private dental care) databases. Because the MarketScan® data are deidentified in compliance with the Health Insurance Portability and Accountability Act of 1996 (HIPAA), and meet limited-use dataset criteria, studies using the data are exempt IRB review.1 All enrollees over 18, with more than 16 months of linked medical, dental and prescribing data from May 1, 2007 through August 31, 2015 were included.

We only included data until August 2015, because in October 2015 the US transitioned from using ICD-9 to ICD-10 diagnosis and procedure codes. This change caused major disruption to the recording of diagnoses and procedures because ICD-10 codes don’t always translate directly into corresponding ICD-9 codes. Indeed, numerous studies using US coding data that spanned the changeover period have reported significant disruption to the recording of the incidence or prevalence of specific diagnoses. Hence, to avoid this disruption and to ensure data integrity and continuity we confined our study to the period before October 2015. Changes to the recording of CPT (medical procedure codes) and CDT (dental procedure codes) after October 2015 caused further disruption that could have affected the data used in this study.

*IE admissions and IE-risk stratification:*

ICD-9 or CPT diagnosis/procedure codes were used to identify individuals as being at high-IE-risk (Table S1) or moderate-IE-risk (Table S2) as defined by the AHA guidelines.2-4 Individuals not so identified were considered at low/unknown-IE-risk.

IE-hospital-admissions were identified using ICD-9 421.0, 421.1 or 421.9, primary or secondary discharge diagnosis codes. Previously described methods were used to ensure single continuous IE-episodes were only counted once.5 After IE-admission, enrollees were considered at high-risk for future IE episodes. New episodes were distinguished from readmissions by only accepting IE-admissions >6 months apart.6, 7

*Invasive Dental-Procedures:*

The American Dental Association (ADA) Code on Dental Procedures and Nomenclature (CDT codes)8 and ICD-9 procedure codes9 were used to classify procedures into; (i) Invasive-dental procedures (IDPs) – those dental procedures that involve manipulation of gingival tissue or the periapical region of the teeth, or perforation of the oral mucosa e.g. dental extractions, oral surgical procedures, scaling (supragingival or subgingival) and endodontic procedures, i.e. those dental procedures that the AHA guidelines recommend ‘should’ be covered by AP.3, 4 When a dental visit included multiple-procedures, the most invasive was ascribed to that visit. We also sub-analysed IDPs using codes specific for periodontal probing, dental prophylaxis (supragingival scaling and/or polishing), sub gingival scaling and/or root planing, extractions

(including surgical extractions), endodontic procedures, surgical-procedures (including biopsies, implant and periodontal surgical procedures) (Table S3 and S4).

Prescription benefits data were used to identify whether each dental procedure was likely to have been AP-covered or not using methodology previously described10 and briefly outlined here. For each patient in the cohort, that patient’s prescription benefits data was searched for antibiotic prophylaxis (AP) prescriptions matching the 2007 AHA recommendations.3 These were identified in the database using the following prescribing criteria (a) mode of antibiotic delivery – oral, (b) antibiotic – amoxicillin, clindamycin, cephalexin, azithromycin or clarithromycin, (c) dosage - 2g for amoxicillin, 600 mg for clindamycin, 2g for cephalexin, 500mg for azithromycin or 500mg for clarithromycin. Our earlier study identified that dentists often prescribed multiple courses of AP cover as a single prescription, in order to ensure that patients had sufficient supplies to cover several invasive dental procedure visits i.e. to avoid the patient having to fill a separate prescription for each invasive dental procedure visit. They also often prescribed at the end of a course of dental treatment so that the patient would have supplies available in advance for a future course of dental treatment. To address these eventualities, we evaluated several different algorithms against the gold standard of the actual prescribing and dental records of 80 patients at high IE-risk, 40 moderate-risk and 40 low-unknown risk patients. The algorithm that best identified when an invasive dental procedure was likely to have been covered by AP included the 3 elements above (a-c) where the number of day’s supply of the antibiotic was −:5 and the time between the prescription fill date and the invasive dental procedure date was −:73 where the number of day’s supply = 1, −:146 where day’s supply = 2, −:219 where day’s supply = 3, −:292 where day’s supply = 4 or −:365 where day’s supply = 5. Using this algorithm had 88% (95% CI 82-92%) sensitivity and 96% (95% CI 94-97%) specificity for identifying when a dental procedure was likely to have been covered by AP29 and this was the algorithm employed in the current study to determine if a dental procedure was likely to have been covered by AP or not.10

*Cohort Study:*

The entire cohort consisted of 9.63 million individuals with linked medical, dental and prescription data. Of these 7.95 million were individuals with employer-provided Commercial/Medicare-Supplemental health cover and 1.68 million were patients with Medicaid cover. Subjects were stratified according to IE-risk (high, moderate or low/unknown) and followed until study completion, expiry of linked-data, or death. For each IE-risk-group, the incidence of IE in the 4 months following IDPs was quantified. IE incidence was compared between different IE risk-groups and different types of IDP. Because AP cover of IDP was used in this population we quantified the level of AP cover of IDP in each risk group of patients (high-risk, moderate- risk and low-risk). Since previous studies have shown high AP efficacy in preventing IE following IDP in these populations,11, 12 we corrected the data on IE incidence following IDP to account for AP use.

Supplementary Appendix Table 1. ICD-9-CM Diagnosis and Procedure Codes, and CPT Outpatient Procedure codes used to identify ‘high risk’ cardiac conditions

| Cardiac Condition | ICD-9-CM Codes (and CPT outpatient procedure codes) |
| --- | --- |
| Previous IE | ICD-9 Diagnostic Code:  4210 acute and subacute bacterial endocarditis  4211 acute and subacute infective endocarditis (in diseases classified elsewhere)  4219 acute endocarditis, unspecified |
| Prosthetic cardiac valve | ICD-9 Procedure Codes:  3505 endovascular replacement of aortic valve 3506 transapical replacement of aortic valve  3507 endovascular replacement of pulmonary valve 3508 transapical replacement of pulmonary valve  3509 endovascular replacement of unspecified heart valve 3520 open and other replacement of unspecified heart valve  3521 open and other replacement of aortic valve with tissue graft 3522 open and other replacement of aortic valve  3523 open and other replacement of mitral valve with tissue graft 3524 open and other replacement of mitral valve  3525 open and other replacement of pulmonary valve with tissue graft 3526 open and other replacement of pulmonary valve  3527 open and other replacement of tricuspid valve with tissue graft 3528 open and other replacement of tricuspid valve  3583 total repair of truncus arteriosus  CPT Procedure Codes:  0256T Implantation of catheter-delivered prosthetic aortic heart valve; endovascular approach 0257T Implantation of catheter-delivered prosthetic aortic heart valve; open thoracic approach (eg,  transapical, transventricular)  0258T Transthoracic cardiac exposure (eg, sternotomy, thoracotomy, subxiphoid) for catheter-delivered aortic valve replacement; without cardiopulmonary bypass  0268T Implantation of catheter-delivered prosthetic pulmonary valve, endovascular approach 0318T Implantation of catheter-delivered prosthetic aortic heart valve, open thoracic approach, (eg,  transapical, other than transaortic)  33361 Transcatheter aortic valve replacement (TAVR/TAVI) with prosthetic valve; percutaneous femoral artery approach  33362 Transcatheter aortic valve replacement (TAVR/TAVI) with prosthetic valve; open femoral artery approach  33363 Transcatheter aortic valve replacement (TAVR/TAVI) with prosthetic valve; open axillary artery approach  33364 Transcatheter aortic valve replacement (TAVR/TAVI) with prosthetic valve; open iliac artery approach  33365 Transcatheter aortic valve replacement (TAVR/TAVI) with prosthetic valve; transaortic approach (eg, median sternotomy, mediastinotomy)  33366 Transcatheter aortic valve replacement (TAVR/TAVI) with prosthetic valve; transapical exposure (eg, left thoracotomy)  33405 Replacement, aortic valve, with cardiopulmonary bypass; with prosthetic valve other than homograft or stentless valve  33406 Replacement, aortic valve, with cardiopulmonary bypass; with allograft valve (freehand) 33410 Replacement, aortic valve, with cardiopulmonary bypass; with stentless tissue valve 33411 Replacement, aortic valve; with aortic annulus enlargement, noncoronary sinus  33412 Replacement, aortic valve; with transventricular aortic annulus enlargement (Konno procedure) 33413 Replacement, aortic valve; by translocation of autologous pulmonary valve with allograft  replacement of pulmonary valve (Ross procedure) 33430 Replacement, mitral valve, with cardiopulmonary bypass  33465 Replacement, tricuspid valve, with cardiopulmonary bypass 33475 Replacement, pulmonary valve  33477 Transcatheter pulmonary valve implantation, percutaneous approach, including pre-stenting of the valve delivery site, when performed  33496 Repair of non-structural prosthetic valve dysfunction with cardiopulmonary bypass (separate procedure) |

|  | ICD-9 Diagnostic Codes:  99602 mechanical complication due to heart valve prosthesis 99671 other complications due to heart valve prosthesis V433 heart valve replaced by other means |
| --- | --- |
| Prosthetic material used for valve repair | ICD-9 Procedure Codes:  3533 annuloplasty  3597 percutaneous mitral valve repair with implant  CPT Procedure Codes:  0343T Transcatheter mitral valve repair percutaneous approach including transseptal puncture when performed; initial prosthesis  0344T Transcatheter mitral valve repair percutaneous approach including transseptal puncture when performed; additional prosthesis (es) during same session (List separately in addition to code for primary procedure)  33391 Valvuloplasty, aortic valve, open, with cardiopulmonary bypass; complex (eg, leaflet extension, leaflet resection, leaflet reconstruction, or annuloplasty)  33418 Transcatheter mitral valve repair, percutaneous approach, including transseptal puncture when performed; initial prosthesis  33419 Transcatheter mitral valve repair, percutaneous approach, including transseptal puncture when performed; additional prosthesis(es) during same session (List separately in addition to code for primary procedure)  33426 Valvuloplasty, mitral valve, with cardiopulmonary bypass; with prosthetic ring  33427 Valvuloplasty, mitral valve, with cardiopulmonary bypass; radical reconstruction, with or without ring  33464 Valvuloplasty, tricuspid valve; with ring insertion  33468 Tricuspid valve repositioning and plication for Ebstein anomaly  33478 Outflow tract augmentation (gusset), with or without commissurotomy or infundibular resection 33600 Closure of atrioventricular valve (mitral or tricuspid) by suture or patch  33602 Closure of semilunar valve (aortic or pulmonary) by suture or patch  33612 Repair of double outlet right ventricle with intraventricular tunnel repair; with repair of right ventricular outflow tract obstruction  33860 Ascending aorta graft, with cardiopulmonary bypass, includes valve suspension, when performed 33861 Ascending aorta graft, with cardiopulmonary bypass, with or without valve suspension; with  coronary reconstruction  33863 Ascending aorta graft, with cardiopulmonary bypass, with aortic root replacement using valved conduit and coronary reconstruction (eg, Bentall)  33864 Ascending aorta graft, with cardiopulmonary bypass with valve suspension, with coronary  reconstruction and valve-sparing aortic root remodeling (eg, David Procedure, Yacoub Procedure) |
| Unrepaired cyanotic congenital heart disease (CHD) | ICD-9 Diagnostic Codes:  7450 common truncus  74510 complete transposition of great vessels 74511 double outlet right ventricle  74519 other transposition of great vessels 7452 tetralogy of Fallot  7453 common ventricle  74560 endocardial cushion defects 7457 cor biloculare  74741 total anomalous pulmonary venous connection |
| CHC in whom a palliative shunt or conduit has been used | ICD-9 Procedure Codes:  3541 enlargement of existing arterial septal defect 3542 creation of septal defect in heart  3591 interatrial transposition of venous return  3592 creation of conduit between right ventricle and pulmonary artery 3593 creation of conduit between left ventricle and aorta  3594 creation of conduit between atrium and pulmonary artery 390 systemic to pulmonary shunt  3921 caval-pulmonary artery anastomosis  CPT Procedure Codes:  33404 Construction of apical-aortic conduit  33606 Anastomosis of pulmonary artery to aorta (Damus-Kaye-Stansel procedure)  33608 Repair of complex cardiac anomaly other than pulmonary atresia with ventricular septal defect by construction or replacement of conduit from right or left ventricle to pulmonary artery  33610 Repair of complex cardiac anomalies (eg, single ventricle with subaortic obstruction) by surgical enlargement of ventricular septal defect |

|  | 33611 Repair of double outlet right ventricle with intraventricular tunnel repair;  33612 Repair of double outlet right ventricle with intraventricular tunnel repair; with repair of right ventricular outflow tract obstruction  33615 Repair of complex cardiac anomalies (eg, tricuspid atresia) by closure of atrial septal defect and anastomosis of atria or vena cava to pulmonary artery (simple Fontan procedure)  33617 Repair of complex cardiac anomalies (eg, single ventricle) by modified Fontan procedure  33619 Repair of single ventricle with aortic outflow obstruction and aortic arch hypoplasia (hypoplastic left heart syndrome) (eg, Norwood procedure)  33697 Complete repair tetralogy of Fallot with pulmonary atresia including construction of conduit from right ventricle to pulmonary artery and closure of ventricular septal defect  33735 Atrial septectomy or septostomy; closed heart (Blalock-Hanlon type operation) 33736 Atrial septectomy or septostomy; open heart with cardiopulmonary bypass 33737 Atrial septectomy or septostomy; open heart, with inflow occlusion  33750 Shunt; subclavian to pulmonary artery (Blalock-Taussig type operation) 33755 Shunt; ascending aorta to pulmonary artery (Waterston type operation) 33762 Shunt; descending aorta to pulmonary artery (Potts-Smith type operation) 33764 Shunt; central, with prosthetic graft  33766 Shunt; superior vena cava to pulmonary artery for flow to 1 lung (classical Glenn procedure) 33767 Shunt; superior vena cava to pulmonary artery for flow to both lungs (bidirectional Glenn  procedure)  33768 Anastomosis, cavopulmonary, second superior vena cava (List separately in addition to primary procedure)  33774 Repair of transposition of the great arteries, atrial baffle procedure (eg, Mustard or Senning type) with cardiopulmonary bypass;  33775 Repair of transposition of the great arteries, atrial baffle procedure (eg, Mustard or Senning type) with cardiopulmonary bypass; with removal of pulmonary band  33776 Repair of transposition of the great arteries, atrial baffle procedure (eg, Mustard or Senning type) with cardiopulmonary bypass; with closure of ventricular septal defect  33777 Repair of transposition of the great arteries, atrial baffle procedure (eg, Mustard or Senning type) with cardiopulmonary bypass; with repair of subpulmonic obstruction  33782 Aortic root translocation with ventricular septal defect and pulmonary stenosis repair (ie, Nikaidoh procedure); without coronary ostium reimplantation  33783 Aortic root translocation with ventricular septal defect and pulmonary stenosis repair (ie, Nikaidoh procedure); with reimplantation of 1 or both coronary ostia  33786 Total repair, truncus arteriosus (Rastelli type operation)  33918 Repair of pulmonary atresia with ventricular septal defect, by unifocalization of pulmonary arteries; without cardiopulmonary bypass  33920 Repair of pulmonary atresia with ventricular septal defect, by construction or replacement of conduit from right or left ventricle to pulmonary artery  92992 Atrial septectomy or septostomy; transvenous method, balloon (eg, Rashkind type) (includes cardiac catheterization)  92993 Atrial septectomy or septostomy; blade method (Park septostomy) (includes cardiac catheterization) |
| --- | --- |
| Completely repaired CHD defect with prosthetic material or device, whether placed by surgery or catheter intervention, during first 6 months after the procedure only. | ICD-9 Procedure Codes:  3550 repair of unspecified septal defect of heart with prosthesis 3551 repair of atrial septal defect with prosthesis, open technique 3552 repair of atrial septal defect with prosthesis, closed technique  3553 repair of ventricular septal defect with prosthesis, open technique 3554 repair of endocardial cushion defect with prosthesis  3555 repair of ventricular septal defect with prosthesis, closed technique 3560 repair of unspecified septal defect of heart with tissue graft  3561 repair of atrial septal defect with tissue graft  3562 repair of ventricular septal defect with tissue graft 3563 repair of endocardial cushion defect with tissue graft  3570 other and unspecified repair of unspecified septal defect of heart 3571 other and unspecified repair of atria septal defect  3572 other and unspecified repair of ventricular septal defect 3573 other and unspecified repair of endocardial cushion defect 3581 total repair of tetralogy of Fallot  3582 total repair of total anomalous pulmonary venous connection 3584 total correction of transposition of great vessels  3598 other operations on septa of heart  CPT Procedure Codes  0166T Transmyocardial transcatheter closure of ventricular septal defect, with implant; without cardiopulmonary bypass |

|  | 0167T Transmyocardial transcatheter closure of ventricular septal defect, with implant; with cardiopulmonary bypass  33545 Repair of postinfarction ventricular septal defect, with or without myocardial resection 33641 Repair atrial septal defect, secundum, with cardiopulmonary bypass, with or without patch  33645 Direct or patch closure, sinus venosus, with or without anomalous pulmonary venous drainage 33647 Repair of atrial septal defect and ventricular septal defect, with direct or patch closure  33660 Repair of incomplete or partial atrioventricular canal (ostium primum atrial septal defect), with or without atrioventricular valve repair  33665 Repair of intermediate or transitional atrioventricular canal, with or without atrioventricular valve repair  33670 Repair of complete atrioventricular canal, with or without prosthetic valve 33675 Closure of multiple ventricular septal defects;  33676 Closure of multiple ventricular septal defects; with pulmonary valvotomy or infundibular resection (acyanotic)  33677 Closure of multiple ventricular septal defects; with removal of pulmonary artery band, with or without gusset  33681 Closure of single ventricular septal defect, with or without patch;  33684 Closure of single ventricular septal defect, with or without patch; with pulmonary valvotomy or infundibular resection (acyanotic)  33688 Closure of single ventricular septal defect, with or without patch; with removal of pulmonary artery band, with or without gusset  33692 Complete repair tetralogy of Fallot without pulmonary atresia;  33694 Complete repair tetralogy of Fallot without pulmonary atresia; with transannular patch  33710 Repair sinus of Valsalva fistula, with cardiopulmonary bypass; with repair of ventricular septal defect 33770 Repair of transposition of the great arteries with ventricular septal defect and subpulmonary  stenosis; without surgical enlargement of ventricular septal defect  33771 Repair of transposition of the great arteries with ventricular septal defect and subpulmonary stenosis; with surgical enlargement of ventricular septal defect  33778 Repair of transposition of the great arteries, aortic pulmonary artery reconstruction (eg, Jatene type);  33779 Repair of transposition of the great arteries, aortic pulmonary artery reconstruction (eg, Jatene type); with removal of pulmonary band  33780 Repair of transposition of the great arteries, aortic pulmonary artery reconstruction (eg, Jatene type); with closure of ventricular septal defect  33781 Repair of transposition of the great arteries, aortic pulmonary artery reconstruction (eg, Jatene type); with repair of subpulmonic obstruction  93580 Percutaneous transcatheter closure of congenital interatrial communication (ie, Fontan fenestration, atrial septal defect) with implant  93581 Percutaneous transcatheter closure of a congenital ventricular septal defect with implant |
| --- | --- |

Notes:

1. Patients at “high-risk” of developing IE were identified by determining whether they had been diagnosed with a “high-risk” condition (ICD-9 codes) or undergone a “high risk” procedure (ICD-9 or CPT procedure codes) at any time before they first developed IE or at any-time for those who did not develop IE during the study period (within the available healthcare records for that individual).
2. Since the AHA guidelines consider patients with congenital heart disease repaired with prosthetic material to be high risk only for the first 6 months after the procedure, they were treated as high-risk for the first 6 months after the procedure only.

Supplementary Appendix Table 2. ICD-9-CM Diagnosis and Procedure Codes, and CPT Outpatient Procedure codes used to identify ‘moderate risk’ cardiac conditions

| Cardiac Condition | ICD-9-CM Codes |
| --- | --- |
| Previous Rheumatic Fever | ICD-9 Diagnostic Codes:  390 rheumatic fever without heart involvement 3910 acute rheumatic pericarditis  3911 acute rheumatic endocarditis 3912 acute rheumatic myocarditis  3918 other acute rheumatic heart disease  3919 acute rheumatic heart disease, unspecified 3920 rheumatic chorea with heart involvement 3929 rheumatic chorea without heart involvement 3941 rheumatic mitral insufficiency  3940 mitral stenosis  3942 mitral stenosis with insufficiency 3949 other unspecified mitral disease 3950 rheumatic aortic stenosis  3951 rheumatic aortic insufficiency  3952 rheumatic aortic stenosis with insufficiency 3959 other and unspecified aortic rheumatic diseases 3960 mitral and aortic stenosis  3961 mitral stenosis and aortic insufficiency 3962 mitral insufficiency and aortic stenosis 3963 mitral insufficiency and aortic insufficiency  3968 multiple involvement of mitral and aortic valves 3969 mitral and aortic valve disease unspecified 3970 diseases of tricuspid valve  3971 rheumatic diseases of pulmonary valve  3979 rheumatic diseases of endocardium, valve unspecified 39890 rheumatic heart disease, unspecified  39899 other rheumatic heart diseases |
| Non-Rheumatic Valve Disease | ICD-9 Diagnostic codes:  4240 mitral valve disorders 4241 aortic valve disorders  4242 tricuspid valve disorders specified as non-rheumatic  4243 pulmonary valve disorders |
| Hypertrophic cardiomyopathy | ICD-9 Diagnostic Codes:  42511 hypertrophic obstructive cardiomyopathy 42518 other hypertrophic cardiomyopathy |
| Congenital valve anomalies | ICD-9 Diagnostic codes:  74600 congenital pulmonary valve anomaly, unspecified 74601 atresia of pulmonary valve, congenital  74602 stenosis of pulmonary valve, congenital  74609 other congenital anomalies of pulmonary valve 7461 tricuspid atresia and stenosis, congenital  7462 Ebstein’s anomaly  7463 congenital stenosis of aortic valve 7464 congenital insufficiency of aortic valve 7465 congenital mitral stenosis  7466 congenital mitral insufficiency 7467 hyperplastic left heart syndrome 74681 sub-aortic stenosis  74683 infundibular pulmonic stenosis  74689 other specified congenital heart anomalies of heart |

Notes: Patients at “moderate risk” of developing IE were identified by determining whether they had been diagnosed with a “moderate risk” condition (ICD-9 codes) at any time before they first developed IE or at any-time for those who did not develop IE during the study period (within the available healthcare records for that individual).

Supplementary Appendix Table 3. Examples of invasive-dental procedures (IDPs)

| Invasive-Dental Procedures (IDPs) – procedures that should be covered by AP |
| --- |
| Dental extractions (including surgical removal of impacted teeth and residual tooth roots) |
| Oral surgery procedures (including biopsies, periodontal surgery, implant surgery and other  oral surgery and maxillofacial procedures involving oral soft tissues or bone) |
| Scaling procedures (including dental prophylaxis, periodontal scaling and root planning, periodontal maintenance and gingival irrigation or delivery of antimicrobial agents into the  diseased gingival crevice) |
| Endodontic treatment (including pulpal debridement, endodontic treatment and re-  treatment, apexification/recalcification, apicectomy and peri-radicular procedures) |

Notes: Based on American Heart Association guidelines.^3,^ ^4^ More extensive details of the dental procedures (including the relevant American Dental Association CDT and ICD-9 procedure codes) used to define invasive-dental procedures (IDPs), and each category of IDP (extractions, oral surgical procedures, scaling and endodontic treatments etc) are provided in Supplemental Tables S3 and S4.

Supplementary Appendix Table 4. CDT and ICD-9 invasive dental procedure (IDP) codes

| Analyses | CDT Codes ICD-9 Codes | |
| --- | --- | --- |
| All Invasive Dental Procedure (IDP) Codes i.e. those procedures that ‘should’ be covered by AP according to the 2007 AHA recommendations | D0180, D0472-4, D1110, D1120, D3221, D3310, D3320, D3330, D3332- 2301, 2309, 2311, 2319,  3, D3346-8, D3351-3, D3410, D3421, D3425-32, D3450, D3460, D3470, 235, 236, 2370-3, 240,  D3910, D3920, D4210-2, D4230-1, D4240-1, D4245, D4249, D4260-1, 2411-2, 242, 2431-2, 2439,  D4263-8, D4270, D4273-8, D4283, D4341-2, D4346, D4355, D4381, 244, 245, 246, 2491, 2499,  D4910, D4921, D6010-3, D6040, D6050, D6080-1, D6100-4, D7111, 2502, 251, 252, 253, 254,  D7140, D7210, D7220, D7230, D7240-1, D7250-1, D7260-1, D7270, 2551, 2559, 2591-4, 2599,  D7272, D7280, D7282-3, D7285-6, D7290-5, D7310-1, D7320-1, D7340, 260, 2612, 2621, 2629-32,  D7350, D7410-5, D7465, D7440-1, D7450-1, D7460-1, D7471-3, D7485, 2641-2, 2649, 270, 271, D7490, D7510-1, D7520-1, D7530, D7540, D7550, D7560, D7610, 2721-4, 2731-2, 2741-3, D7630, D7671, D7710, D7730, D7770, D7941, D7943-50, D7952-3, 2749, 2751-7, 2759, 2761-4,  D7955, D7960, D7963, D7970-2, D7981-3, D7991, D7996-8 2769, 2771-3, 2779, 2791-2,  2799, 9654 | |
| Codes For Specific Types of IDP | | |
| Periodontal Probing | D0180 |  |
| Dental Prophylaxis and Scaling | D1110, D1120, D4341, D4342, D4346, D4355, D4381, D4910, D4921, D4999 | 9654 |
| - Dental prophylaxis | D1110, D1120 |  |
| - Subgingival scaling and/or root planing | D4341, D4342, D4346, D4355, D4381, D4910, D4921, D4999 | 9654 |
| Extractions (including surgical extractions) | D7111, D7140, D7210, D7220, D7230, D7240, D7241, D7250, D7251 | 2301, 2309, 2311, 2319, |
| Endodontic Procedures | D3221, D3310, D3320, D3330, D3332-3, D3346-8, D3351-3, D3410, D3421, D3425-32, D3450, D3460, D3470, D3910, D3920, | 2370-3, |
| Surgical Procedures (including oral surgical procedures, periodontal surgery procedures and biopsies) | D0472-4, D4210-2, D4230-1, D4240-1, D4245, D4249, D4260-1, D4263-8, D4270, D4273-8, D4283, D7260-1, D7270, D7272, D7280, D7282-3, D7285-6, D7290-5, D7310-1, D7320-1, D7340, D7350,  D7410-5, D7465, D7440-1, D7450-1, D7460-1, D7471-3, D7485, D7490, D7510-1, D7520-1, D7530, D7540, D7550, D7560, D7610, D7630, D7671, D7710, D7730, D7770, D7941, D7943-50, D7952-3, D7955, D7960, D7963, D7970-2, D7981-3, D7991, D7996-8 | 240, 2411-2, 242, 2431-2,  2439, 244, 245, 246, 2491,  2499, 2502, 251, 252, 253,  254, 2551, 2559, 2591-4,  2599, 260, 2612, 2621,  2629-32, 2641-2, 2649, 270,  271, 2721-4, 2731-2, 2741-  3, 2749, 2751-7, 2759,  2761-4, 2769, 2771-3, 2779,  2791-2, 2799, |
| Restorative Procedures – likely to involve gingival manipulation | D2160, D2161, D2332, D2335, D2393, D2394, D2530.D2630, D2652, D2710, D2712, D2720, D2721, D2722, D2740, D2750, D2751, D2752, D2780-83, D2790-94, D2799 |  |

Supplementary Appendix Table 5. Definitions for ADA Procedure Codes used in this study

| ADA CDT-2017 CODE ON DENTAL PROCEDURES AND NOMENCLATURE | |
| --- | --- |
| D0100-D0999 DIAGNOSTIC | |
| CLINICAL ORAL EVALUATIONS | |
| D0180 | Comprehensive periodontal evaluation - new or established patient |
| ORAL PATHOLOGY LABORATORY | |
| D0472 | Accession of tissue, gross examination, preparation and transmission of written report |
| D0473 | Accession of tissue, gross and microscopic examination, preparation and transmission of written report |
| D0474 | Accession of tissue, gross and microscopic examination, including assessment of surgical margins for presence of disease, preparation and transmission of written report |
| D1000-D1999 PREVENTIVE | |
| DENTAL PROPHYLAXIS | |
| D1110 | Prophylaxis - adult |
| D1120 | Prophylaxis - child |
| D2000-D2999 RESTORATIVE | |
| AMALGAM RESTORATIONS (INCLUDING POLISHING) | |
| D2160 | Amalgam - three surfaces, primary or permanent |
| D2161 | Amalgam - four or more surfaces, primary or permanent |
| RESIN-BASED COMPOSITE RESTORATIONS - DIRECT | |
| D2332 | Resin-based composite - three surfaces, anterior |
| D2335 | Resin-based composite - four or more surfaces or involving incisal angle (anterior) |
| D2393 | Resin-based composite - three surfaces, posterior |
| D2394 | Resin-based composite - four or more surfaces, posterior |
| INLAY/ONLAY RESTORATIONS | |
| D2530 | Inlay - metallic - three or more surfaces |
| D2630 | Inlay - porcelain/ceramic - three or more surfaces |
| D2652 | Inlay - resin-based composite - three or more surfaces |
| CROWNS | |
| D2710 | Crown - resin-based composite (indirect) |
| D2712 | Crown - ¾ resin-based composite (indirect) |
| D2720 | Crown - resin with high noble metal |

| D2721 | Crown - resin with predominantly base metal |
| --- | --- |
| D2722 | Crown - resin with noble metal |
| D2740 | Crown - porcelain/ceramic substrate |
| D2750 | Crown - porcelain fused to high noble metal |
| D2751 | Crown - porcelain fused to predominantly base metal |
| D2752 | Crown - porcelain fused to noble metal |
| D2780 | Crown - 3/4 cast high noble metal |
| D2781 | Crown - 3/4 cast predominantly base metal |
| D2782 | Crown - 3/4 cast noble metal |
| D2783 | Crown - 3/4 porcelain/ceramic |
| D2790 | Crown - full cast high noble metal |
| D2791 | Crown - full cast predominantly base metal |
| D2792 | Crown - full cast noble metal |
| D2794 | Crown - titanium |
| D2799 | Provisional crown– further treatment or completion of diagnosis necessary prior to final impression |
| D3000-D3999 ENDODONTICS | |
| PULPOTOMY | |
| D3221 | Pulpal debridement, primary and permanent teeth |
| ENDODONTIC THERAPY | |
| D3310 | Endodontic therapy, anterior tooth (excluding final restoration) |
| D3320 | Endodontic therapy, bicuspid tooth (excluding final restoration) |
| D3330 | Endodontic therapy, molar (excluding final restoration) |
| D3332 | Incomplete endodontic therapy; inoperable, unrestorable or fractured tooth |
| D3333 | Internal root repair of perforation defects |
| ENDODONTIC RETREATMENT | |
| D3346 | Retreatment of previous root canal therapy - anterior |
| D3347 | Retreatment of previous root canal therapy - bicuspid |
| D3348 | Retreatment of previous root canal therapy - molar |
| APEXIFICATION/RECALCIFICATION | |
| D3351 | Apexification/recalcification – initial visit (apical closure/calcific repair of perforations, root resorption, etc.) |
| D3352 | Apexification/recalcification - interim medication replacement |

| D3353 | Apexification/recalcification - final visit (includes completed root canal therapy - apical closure/calcific repair of perforations, root resorption, etc.) |
| --- | --- |
| APICOECTOMY/PERIRADICULAR SERVICES | |
| D3410 | Apicoectomy - anterior |
| D3421 | Apicoectomy - bicuspid (first root) |
| D3425 | Apicoectomy - molar (first root) |
| D3426 | Apicoectomy (each additional root) |
| D3427 | Periradicular surgery without apicoectomy |
| D3428 | Bone graft in conjunction with periradicular surgery – per tooth, single site |
| D3429 | Bone graft in conjunction with periradicular surgery – each additional contiguous tooth in the same surgical site |
| D3430 | Retrograde filling - per root |
| D3431 | Biologic materials to aid in soft and osseous tissue regeneration in conjunction with periradicular surgery |
| D3432 | Guided tissue regeneration, resorbable barrier, per site, in conjunction with periradicular surgery |
| D3450 | Root amputation - per root |
| D3460 | Endodontic endosseous implant |
| D3470 | Intentional re-implantation (including necessary splinting) |
| OTHER ENDODONTIC PROCEDURES | |
| D3910 | Surgical procedure for isolation of tooth with rubber dam |
| D3920 | Hemisection (including any root removal), not including root canal therapy |
| D4000-D4999 PERIODONTICS | |
| SURGICAL SERVICES (INCLUDING USUAL POSTOPERATIVE CARE) | |
| D4210 | Gingivectomy or gingivoplasty - four or more contiguous teeth or tooth bounded spaces per quadrant |
| D4211 | Gingivectomy or gingivoplasty - one to three contiguous teeth or tooth bounded spaces per quadrant |
| D4212 | Gingivectomy or gingivoplasty to allow access for restorative procedure, per tooth |
| D4230 | Anatomical crown exposure - four or more contiguous teeth per quadrant |
| D4231 | Anatomical crown exposure - one to three teeth per quadrant |
| D4240 | Gingival flap procedure, including root planing - four or more contiguous teeth or tooth bounded spaces per quadrant |
| D4241 | Gingival flap procedure, including root planing - one to three contiguous teeth or tooth bounded spaces per quadrant |
| D4245 | Apically positioned flap |
| D4249 | Clinical crown lengthening - hard tissue |

| D4260 | Osseous surgery (including elevation of a full thickness flap and closure) - four or more contiguous teeth or tooth bounded spaces per quadrant |
| --- | --- |
| D4261 | Osseous surgery (including elevation of a full thickness flap and closure) - one to three contiguous teeth or tooth bounded spaces per quadrant |
| D4263 | Bone replacement graft - retained natural tooth - first site in quadrant |
| D4264 | Bone replacement graft - retained natural tooth - each additional site in quadrant |
| D4265 | Biologic materials to aid in soft and osseous tissue regeneration |
| D4266 | Guided tissue regeneration - resorbable barrier, per site |
| D4267 | Guided tissue regeneration - nonresorbable barrier, per site (includes membrane removal) |
| D4268 | Surgical revision procedure, per tooth |
| D4270 | Pedicle soft tissue graft procedure |
| D4273 | Autogenous connective tissue graft procedure (including donor and recipient surgical sites) first tooth, implant or edentulous tooth position in graft |
| D4283* | Autogenous connective tissue graft procedure (including donor and recipient surgical sites) - each additional contiguous tooth, implant or edentulous tooth position in same graft site |
| D4275* | Non-autogenous connective tissue graft (including recipient site and donor material) first tooth, implant, or edentulous tooth position in graft |
| D4285* | Non-autogenous connective tissue graft (including recipient surgical site and donor material) - each additional contiguous tooth, implant, or edentulous tooth position in same graft site |
| D4274 | Mesial/distal wedge procedure, single tooth (when not performed in conjunction with surgical procedures in the same anatomical area) |
| D4276 | Combined connective tissue and double pedicle graft, per tooth |
| D4277 | Free soft tissue graft procedure (including recipient and donor surgical sites) first tooth, implant or edentulous tooth position in graft |
| D4278 | Free soft tissue graft procedure (including recipient and donor surgical sites) each additional contiguous tooth, implant or edentulous tooth position in same graft site |
| NON-SURGICAL PERIODONTAL SERVICE | |
| D4341 | Periodontal scaling and root planing - four or more teeth per quadrant |
| D4342 | Periodontal scaling and root planing - one to three teeth per quadrant |
| D4346 | Scaling in the presence of generalized moderate or severe gingival inflammation - full mouth, after oral evaluation |
| D4355 | Full mouth debridement to enable comprehensive evaluation and diagnosis |
| D4381 | Localized delivery of antimicrobial agents via controlled release vehicle into diseased crevicular tissue, per tooth |
| OTHER PERIODONTAL SERVICES | |
| D4910 | Periodontal maintenance |

| D4921 | Gingival irrigation – per quadrant |
| --- | --- |
| D4999 | Unspecified periodontal procedure, by report |
| D6000-D6199 IMPLANT SERVICES | |
| SURGICAL SERVICES | |
| D6010 | Surgical placement of implant body: endosteal implant |
| D6011 | Second stage implant surgery |
| D6012 | Surgical placement of interim implant body for transitional prosthesis: endosteal implant |
| D6013 | Surgical placement of mini implant |
| D6040 | Surgical placement: eposteal implant |
| D6050 | Surgical placement: transosteal implant |
| D6100* | Implant removal, by report |
| D6101* | Debridement of a peri-implant defect or defects surrounding a single implant, and surface cleaning of the exposed implant surfaces, including flap entry and closure |
| D6102* | Debridement and osseous contouring of a peri-implant defect or defects surrounging a single implant and includes surface cleaning of the exposed implant surfaces, including flap entry and closure |
| D6103* | Bone graft for repair of peri-implant defect – does not include flap entry and closure |
| D6104* | Bone graft at time of implant placement |
| OTHER IMPLANT SERVICES | |
| D6080 | Implant maintenance procedures when prostheses are removed and reinserted, including cleansing of prostheses and abutments |
| D6081 | Scaling and debridement in the presence of inflammation or mucositis of a single implant, including cleaning of the implant surfaces, without flap entry and closure |
| D7000-D7999 ORAL & MAXILLOFACIAL SURGERY | |
| EXTRACTIONS (INCLUDES LOCAL ANESTHESIA, SUTURING, IF NEEDED, AND ROUTINE POSTOPERATIVE CARE) | |
| D7111 | Extraction, coronal remnants - deciduous tooth |
| D7140 | Extraction, erupted tooth or exposed root (elevation and/or forceps removal) |
| D7210 | Extraction, erupted tooth requiring removal of bone and/or sectioning of tooth, and including elevation of mucoperiosteal flap if indicated |
| D7220 | Removal of impacted tooth - soft tissue |
| D7230 | Removal of impacted tooth - partially bony |
| D7240 | Removal of impacted tooth - completely bony |
| D7241 | Removal of impacted tooth - completely bony, with unusual surgical complications |

| D7250 | Removal of residual tooth roots (cutting procedure) |
| --- | --- |
| D7251 | Coronectomy – intentional partial tooth removal |
| OTHER SURGICAL PROCEDURES | |
| D7260 | Oroantral fistula closure |
| D7261 | Primary closure of a sinus perforation |
| D7270 | Tooth reimplantation and/or stabilization of accidentally evulsed or displaced tooth |
| D7272 | Tooth transplantation (includes re-implantation from one site to another and splinting and/or stabilization) |
| D7280 | Exposure of an unerupted tooth |
| D7282 | Mobilization of erupted or malpositioned tooth to aid eruption |
| D7285 | Incisional biopsy of oral tissue - hard (bone, tooth) |
| D7286 | Incisional biopsy of oral tissue - soft |
| D7290 | Surgical repositioning of teeth |
| D7292 | Placement of temporary anchorage device [screw retained plate] requiring flap; includes device removal |
| D7293 | Placement of temporary anchorage device requiring flap; includes device removal |
| ALVEOLOPLASTY - PREPARATION OF RIDGE | |
| D7310 | Alveoloplasty in conjunction with extractions - four or more teeth or tooth spaces, per quadrant |
| D7311 | Alveoloplasty in conjunction with extractions - one to three teeth or tooth spaces, per quadrant |
| D7320 | Alveoloplasty not in conjunction with extractions - four or more teeth or tooth spaces, per quadrant |
| D7321 | Alveoloplasty not in conjunction with extractions - one to three teeth or tooth spaces, per quadrant |
| VESTIBULOPLASTY | |
| D7340 | Vestibuloplasty - ridge extension (secondary epithelialization) |
| D7350 | Vestibuloplasty - ridge extension (including soft tissue grafts, muscle reattachment, revision of soft tissue attachment and management of hypertrophied and hyperplastic tissue) |
| EXCISION OF SOFT TISSUE LESIONS | |
| D7410 | Excision of benign lesion up to 1.25 cm |
| D7411 | Excision of benign lesion greater than 1.25 cm |
| D7412 | Excision of benign lesion, complicated |
| D7413 | Excision of malignant lesion up to 1.25 cm |
| D7414 | Excision of malignant lesion greater than 1.25 cm |

| D7415 | Excision of malignant lesion, complicated |
| --- | --- |
| D7465* | Destruction of lesion(s) by physical or chemical method, by report |
| EXCISION OF INTRA-OSSEOUS LESIONS | |
| D7440 | Excision of malignant tumor - lesion diameter up to 1.25 cm |
| D7441 | Excision of malignant tumor - lesion diameter greater than 1.25 cm |
| D7450 | Removal of benign odontogenic cyst or tumor - lesion diameter up to 1.25 cm |
| D7451 | Removal of benign odontogenic cyst or tumor - lesion diameter greater than 1.25 cm |
| D7460 | Removal of benign nonodontogenic cyst or tumor - lesion diameter up to 1.25 cm |
| D7461 | Removal of benign nonodontogenic cyst or tumor - lesion diameter greater than 1.25 cm |
| EXCISION OF BONE TISSUE | |
| D7471 | Removal of lateral exostosis (maxilla or mandible) |
| D7472 | Removal of torus palatinus |
| D7473 | Removal of torus mandibularis |
| D7485 | Reduction of osseous tuberosity |
| D7490 | Radical resection of maxilla or mandible |
| SURGICAL INCISION | |
| D7510 | Incision and drainage of abscess - intraoral soft tissue |
| D7511 | Incision and drainage of abscess - intraoral soft tissue - complicated (includes drainage of multiple fascial spaces) |
| D7520 | Incision and drainage of abscess - extraoral soft tissue |
| D7521 | Incision and drainage of abscess - extraoral soft tissue - complicated (includes drainage of multiple fascial spaces) |
| D7530 | Removal of foreign body from mucosa, skin, or subcutaneous alveolar tissue |
| D7540 | Removal of reaction producing foreign bodies, musculoskeletal system |
| D7550 | Partial ostectomy/sequestrectomy for removal of non-vital bone |
| D7560 | Maxillary sinusotomy for removal of tooth fragment or foreign body |
| TREATMENT OF CLOSED FRACTURES | |
| D7610 | Maxilla - open reduction (teeth immobilized, if present) |
| D7630 | Mandible - open reduction (teeth immobilized, if present) |
| D7671 | Alveolus - open reduction, may include stabilization of teeth |
| TREATMENT OF OPEN FRACTURES | |
| D7710 | Maxilla - open reduction |

| D7730 | Mandible - open reduction |
| --- | --- |
| D7770 | Alveolus - open reduction stabilization of teeth |
| OTHER REPAIR PROCEDURES | |
| D7970 | Excision of hyperplastic tissue - per arch |
| D7971 | Excision of pericoronal gingiva |
| D7996 | Implant-mandible for augmentation purposes (excluding alveolar ridge), by report |

Appendix Table 6. Unadjusted (raw) IE incidence within 4 months of different invasive dental procedures (IDP)

| **Patients with Commercial/Medicare Supplemental Cover** | | | | | | | | | | | | | | | | |
| --- | --- | --- | --- | --- | --- | --- | --- | --- | --- | --- | --- | --- | --- | --- | --- | --- |
| High-Risk of IE | | | | | Moderate-Risk of IE | | | | Low-Risk of IE | | |  | All Patients | | | |
| Type of procedure | Procedu res (n) | IE  (n) | IE/million procedur es | High v Low OR, 95%CI, p | Procedures (n) | IE  (n) | IE/million procedur es | Mod v Low OR, 95%CI, p | Procedures (n) | IE  (n) | IE/m proc es | llion edur | Procedures (n) | | IE (n) | IE/million procedure s |
| Periodontal probing | 1,432 | 2 | 1,397 | 69.128, 14.348-333.046,  p<0.0001 | 25,981 | 2 | 77 | 3.805, 0.790-18.318,  p<0.05 | 345,991 | 7 |  | 20 | 373,404 |  | 11 | 29 |
| All dental prophylaxis/scaling | 161,839 | 166 | 1,026 | 79.670, 66.578-95.336,  p<0.0001 | 2,567,587 | 223 | 87 | 6.740, 5.731-7.926,  p<0.0001 | 32,899,901 | 424 |  | 13 | 35,472,548 |  | 810 | 23 |
| -Prophylaxis | 136,574 | 143 | 1,047 | 83.950, 69.116-101.968,  p<0.0001 | 2,168,556 | 170 | 78 | 6.279, 5.229-7.541,  p<0.0001 | 28,193,407 | 352 |  | 12 | 30,498,537 |  | 665 | 22 |
| **-**Sub-gingival scaling/root planing | 25,265 | 23 | 910 | 59.561, 37.241-95.259,  p<0.0001 | 399,031 | 53 | 133 | 8.683, 6.090-12.381,  p<0.0001 | 4,706,494 | 72 |  | 15 | 5,130,790 |  | 148 | 29 |
| Extractions | 11,483 | 61 | 5,312 | 162.131, 114.116-  230.349, p<0.0001 | 168,278 | 53 | 315 | 9.565, 6.646-13.765,  p<0.0001 | 1,942,999 | 64 |  | 33 | 2,122,760 |  | 178 | 84 |
| Endodontic treatment | 6,621 | 5 | 755 | 36.292, 14.009-94.016,  p<0.0001 | 113,780 | 12 | 105 | 5.065, 2.576-9.961,  p<0.0001 | 1,344,624 | 28 |  | 21 | 1,465,025 |  | 45 | 31 |
| Oral Ssrgery* | 3,628 | 28 | 7,718 | 286.646, 158.415-  518.674, p<0.0001 | 64,663 | 13 | 201 | 7.411, 3.631-15.125,  p<0.0001 | 663,398 | 18 |  | 27 | 731,689 |  | 59 | 81 |
| Restorative rocedures^#^ | 46,143 | 51 | 1,105 | 69.837, 50.662-96.272,  p<0.0001 | 717,454 | 66 | 92 | 5.807, 4.332-7.784,  p<0.0001 | 8,773,341 | 139 |  | 16 | 9,536,938 |  | 256 | 27 |
| Total | 231,146 | 313 | 1,354 | 91.666, 80.175-104.804,  p<0.0001 | 3,657,743 | 369 | 101 | 6.821, 6.009-7.742,  p<0.0001 | 45,970,254 | 680 |  | 15 | 49,702,364 |  | 1,359 | 27 |
| **Patients with Medicaid Cover** | | | | | | | | | | | | | | | | |
| High-Risk of IE Moderate-Risk of IE | | | | | | | | | Low-Risk of IE | | |  |  | All Patients | |  |

| Type of procedure | Procedu res (n) | IE  (n) | IE/million procedur es | High v Low OR, 95%CI, p | Procedures (n) | IE  (n) | IE/million procedur es | Mod v Low OR, 95%CI, p | Procedures (n) | IE  (n) | IE/million procedur es | Procedures (n) | | IE (n) | IE/million procedure s |
| --- | --- | --- | --- | --- | --- | --- | --- | --- | --- | --- | --- | --- | --- | --- | --- |
| Periodontal probing | 22 | 0 | 0 | nc | 89 | 0 | 0 | nc | 1,934 | 0 | 0 | 2,045 | | 0 | 0 |
| All dental prophylaxis/scaling | 7,091 | 15 | 2,115 | 126.763, 66.804-240.534,  p<0.0001 | 59,698 | 13 | 218 | 13.025, 6.663-25.459,  p<0.0001 | 1,494,978 | 25 | 17 | 1,561,767 | | 53 | 34 |
| -Prophylaxis | 6,192 | 10 | 1,615 | 118.785, 54.810-257.422,  p<0.0001 | 51,186 | 9 | 176 | 12.914, 5.801-28.746,  p<0.0001 | 1,321,783 | 18 | 14 | 1,379,161 | | 37 | 27 |
| -Sub-gingival scaling/root planing | 899 | 5 | 5,562 | 138.528, 43.884-437.289,  p<0.0001 | 8,512 | 4 | 470 | 11.632, 3.404-39.742,  p<0.0001 | 173,195 | 7 | 40 | 182,606 | | 16 | 88 |
| Extractions | 6,029 | 55 | 9,123 | 113.674, 80.704-160.113,  p<0.0001 | 72,861 | 54 | 741 | 9.158, 6.495-12.912,  p<0.0001 | 1,012,541 | 82 | 81 | 1,091,431 | | 191 | 175 |
| Endodontic treatment | 403 | 1 | 2,481 | 275.908, 17.228-  4,418.716, p<0.0001 | 3,548 | 2 | 564 | 62.558, 5.671-690.061,  p<0.0001 | 110,916 | 1 | 9 | 114,867 | | 4 | 35 |
| Oral surgery* | 756 | 17 | 22,487 | 96.829, 51.209-183.092,  p<0.0001 | 7,015 | 10 | 1,426 | 6.009, 2.844-12.694,  p<0.0001 | 92,625 | 22 | 238 | 99,946 | | 49 | 490 |
| Restorative procedures^#^ | 3,714 | 9 | 2,423 | 80.164,37-802-169.998,  p<0.0001 | 35,248 | 11 | 312 | 10.302, 5.129-20.694,  p<0.0001 | 924,056 | 28 | 30 | 963,018 | | 48 | 50 |
| Total | 18,015 | 97 | 5,384 | 124.611,96.733-160.523,  p<0.0001 | 178,459 | 90 | 504 | 11.614, 8.965-15.046,  p<0.0001 | 3,637,050 | 158 | 43 | 3,833,074 | | 345 | 90 |
| **All Patients** | | | | | | | | | | | | | | | |
| High-Risk of IE Moderate-Risk of IE | | | | | | | | | Low-Risk of IE | | |  | All Patients | |  |
| Type of procedure | Procedu res (n) | IE  (n) | IE/million procedur es | High v Low OR, 95%CI, p | Procedures (n) | IE  (n) | IE/million procedur es | Mod v Low OR, 95%CI, p | Procedures (n) | IE  (n) | IE/million procedur es | Procedures (n) |  | IE (n) | IE/million procedure s |
| Periodontal probing | 1,454 | 2 | 1,376 | 68.461, 14.210-329.829,  p<0.0001 | 26,070 | 2 | 77 | 3.813, 0.792-18.357,  p<0.05 | 347,925 | 7 | 20 | 375,449 |  | 11 | 29 |

| All dental prophylaxis/scaling | 168,930 | 181 | 1,071 | 82164, 69.136-97.646,  p<0.0001 | 2,627,285 | 236 | 90 | 6.882, 5.878-8.056,  p<0.0001 | 34,394,879 | 449 | 13 | 37,034,315 | 863 | 23 |
| --- | --- | --- | --- | --- | --- | --- | --- | --- | --- | --- | --- | --- | --- | --- |
| -Prophylaxis | 142,766 | 153 | 1,072 | 85.580, 70.880-103.328,  p<0.0001 | 2,219,742 | 179 | 81 | 6.433, 5.382-7.690,  p<0.0001 | 29,515,190 | 370 | 13 | 31,877,698 | 702 | 22 |
| -Sub-gingival scaling/root planing | 26,164 | 28 | 1,070 | 66.172, 42.992-101.850,  p<0.0001 | 407,543 | 57 | 140 | 8.640, 6.146-12.147,  p<0.0001 | 4,879,689 | 79 | 16 | 5,313,396 | 164 | 31 |
| Extractions | 17,512 | 116 | 6,624 | 134.981, 105-731-  172.322, p<0.0001 | 241,139 | 107 | 444 | 10.154, 7.909-13.037,  p<0.0001 | 2,955,540 | 146 | 49 | 3,214,191 | 369 | 115 |
| Endodontic treatment | 7,024 | 6 | 854 | 42.910, 17.810-103.384,  p<0.0001 | 117,328 | 14 | 119 | 5.990, 3.165-11.335,  p<0.0001 | 1,455,540 | 29 | 20 | 1,579,892 | 49 | 31 |
| Oral surgery* | 4,384 | 45 | 10,265 | 196.009, 127.894-300.4,  p<0.0001 | 71,678 | 23 | 321 | 6.066, 3.632-10.132,  p<0.0001 | 756,023 | 40 | 53 | 831,635 | 108 | 130 |
| Restorative rocedures^#^ | 49,857 | 60 | 1,203 | 69.965, 52.084-93.984,  p<0.0001 | 752,702 | 77 | 102 | 5.941, 4.535-7.782,  p<0.0001 | 9,697,397 | 167 | 17 | 10,499,956 | 304 | 29 |
| Total | 249,161 | 410 | 1,646 | 97.569, 86.693-109.810,  p<0.0001 | 3,836,202 | 459 | 120 | 7.084, 6.322-7.938,  p<0.0001 | 49,607,304 | 838 | 17 | 53,535,438 | 1,704 | 32 |

Abbreviations: IE = infective endocarditis, OR = odds ratio. * Includes biopsies, implant and periodontal surgery. ^#^Restorative procedures likely to involve gingival manipulation.

*Supplementary References:*

1. US Department for Health and Human Services. Health Insurance Portability and Accountability Act 1996: US Department for Health and Human Services; 1996.
2. Dajani AS, Taubert KA, Wilson W, et al. Prevention of bacterial endocarditis. Recommendations by the American Heart Association. *Circulation.* 1997;96:358-366.
3. Wilson W, Taubert KA, Gewitz M, et al. Prevention of infective endocarditis: guidelines from the American Heart Association: a guideline from the American Heart Association Rheumatic Fever, Endocarditis, and Kawasaki Disease Committee, Council on Cardiovascular Disease in the Young, and the Council on Clinical Cardiology, Council on Cardiovascular Surgery and Anesthesia, and the Quality of Care and Outcomes Research Interdisciplinary Working Group. *Circulation.* 2007;116:1736-1754.
4. Wilson WR, Gewitz M, Lockhart PB, et al. Prevention of Viridans Group Streptococcal Infective Endocarditis: A Scientific Statement From the American Heart Association. *Circulation.* 2021;143:e963-e978.
5. Thornhill MH, Dayer MJ, Forde JM, et al. Impact of the NICE guideline recommending cessation of antibiotic prophylaxis for prevention of infective endocarditis: before and after study. *BMJ.* 2011;342:d2392.
6. Chu VH, Sexton DJ, Cabell CH, et al. Repeat infective endocarditis: differentiating relapse from reinfection. *Clinical infectious diseases : an official publication of the Infectious Diseases Society of America.* 2005;41:406-409.
7. Thornhill MH, Gibson TB, Cutler E, et al. Antibiotic Prophylaxis and Incidence of Endocarditis Before and After the 2007 AHA Recommendations. *J Am Coll Cardiol.* 2018;72:2443-2454.
8. American Dental Association (ADA). Code on Dental Procedures and Nomenclature (CDT Code): American Dental Association (ADA),; 2019.
9. Centers for Disease Control and Prevention (CDC). International Classification of Diseases,Ninth Revision, Clinical Modification (ICD-9-CM): Centers for Disease Control and Prevention (CDC),; 2019.
10. Thornhill MH, Gibson TB, Durkin MJ, et al. Prescribing of antibiotic prophylaxis to prevent infective endocarditis. *J Am Dent Assoc.* 2020;151:835-845 e831.
11. Thornhill MH, Gibson TB, Yoon F, et al. Antibiotic Prophylaxis Against Infective Endocarditis Before Invasive Dental Procedures. *J Am Coll Cardiol.* 2022;80:1029-1041.
12. Thornhill MH, Gibson TB, Yoon F, et al. Endocarditis, invasive dental procedures, and antibiotic prophylaxis efficacy in US Medicaid patients. *Oral diseases.* 2024;30:1591-1605.

25
